# Dihydroartemisinin Sensitizes Esophageal Squamous Cell Carcinoma to Cisplatin by Inhibiting Sonic Hedgehog Signaling

**DOI:** 10.3389/fcell.2020.596788

**Published:** 2020-12-10

**Authors:** Wei Cui, Tingting Fang, Zhaoheng Duan, Dongfang Xiang, Yanxia Wang, Mengsi Zhang, Fangzheng Zhai, Xiang Cui, Lang Yang

**Affiliations:** ^1^Department of Pharmacology, Shenyang Pharmaceutical University, Shenyang, China; ^2^Institute of Pathology and Southwest Cancer Center, Southwest Hospital, Third Military Medical University (Army Medical University), Chongqing, China; ^3^Department of Breast and Thyroid Surgery, Southwest Hospital, Third Military Medical University (Army Medical University), Chongqing, China; ^4^Department of Gastroenterology, The Seventh Medical Center, Chinese PLA General Hospital, Beijing, China

**Keywords:** cisplatin resistance, esophageal squamous cell carcinoma, Shh pathway, dihydroartemisinin, cancer stem cell

## Abstract

Platinum-based regimens have been routinely used in the clinical treatment of patients with esophageal squamous cell carcinoma (ESCC). However, administration of these drugs is frequently accompanied by drug resistance. Revealing the underlying mechanisms of the drug resistance and developing agents that enhance the sensitivity to platinum may provide new therapeutic strategies for the patients. In the present study, we found that the poor outcome of ESCC patients receiving platinum-based regimens was associated with co-expression of Shh and Sox2. The sensitivity of ESCC cell lines to cisplatin was related to their activity of Shh signaling. Manipulating of Shh expression markedly changed the sensitivity of ESCC cells to platinum. Continuous treatment with cisplatin resulted in the activation of Shh signaling and enhanced cancer stem cell-like phenotypes in ESCC cells. Dihydroartemisinin (DHA), a classic antimalarial drug, was identified as a novel inhibitor of Shh pathway. Treatment with DHA attenuated the cisplatin-induced activation of the Shh pathway in ESCC cells and synergized the inhibitory effect of cisplatin on proliferation, sphere and colony formation of ALDH-positive ESCC cells *in vitro* and growth of ESCC cell-derived xenograft tumors *in vivo*. Taken together, these results demonstrate that the Shh pathway is an important player in cisplatin-resistant ESCC and DHA acts as a promising therapeutic agent to sensitize ESCC to cisplatin treatment.

## Introduction

Esophageal cancer with esophageal squamous cell carcinoma (ESCC) as its main histological type is the eighth most common form of cancer worldwide, and has been demonstrated to be one of the most difficult malignancies to be cured (Arnold et al., 2015). Platinum-based chemotherapy is commonly used for the treatment of patients with unresectable ESCC (Nakajima and Kato, 2013; Rubenstein and Shaheen, 2015). However, the treatment efficacy is diminished duo to the occurrence of platinum resistance (Galanski, 2006; Aichler et al., 2014). Insight into the molecular underpinnings of platinum resistance would benefit the development of novel therapeutic strategies for ESCC patients. Recently, several molecules or signaling pathways have been reported to be involved in the platinum resistance, including sirtuin type 1 (SIRT1) (Cao et al., 2015), multidrug resistance protein 2 (MRP2) (Yamasaki et al., 2011), 14-3-3σ (Lai et al., 2016), leptin and AKT signaling (Bain et al., 2014; Li et al., 2014). Nevertheless, none of them has been used as an indicator to predict the response to platinum or a target to sensitize cancer cells to platinum for patients with ESCC in clinical practice.

Sonic hedgehog (Shh) signaling plays an important role in embryonic development, cell proliferation, tissue polarity, and carcinogenesis (Merchant, 2012; Athar et al., 2014). Once Shh protein binds to its receptor patched 1 (PTCH1), smoothened (SMO) is released from the inhibitory combination of PTCH1 and activates downstream Gli transcriptional factors (Glis). So the target genes of Glis were activated, including *SOX2* (Rimkus et al., 2016) and *PTCH1* (Xie et al., 1998). It has been well demonstrated that the activation of Shh signaling was involved in the development and progression of many types of cancer (Cui et al., 2010; Yoo et al., 2011; Wang et al., 2012; Duan et al., 2015), including ESCC (Berman et al., 2003; Yang et al., 2012). Moreover, the critical roles of Shh pathway in maintaining cancer stem cells (CSCs) and drug resistance have also been well-illustrated (Chase et al., 2010; Zahreddine et al., 2014; Rimkus et al., 2016). Thus, Shh signaling might also been involved in platinum resistance in ESCC.

Dihydroartemisinin (DHA) is a derivative of artemisinin isolated from a traditional Chinese medicine *Artemisia annua*, and has been used as classical antimalarial drug worldwide. In the recent years, DHA has also been reported to have anti-cancer properties in several types of cancer, including ESCC (Efferth, 2017a; Luo et al., 2019). Moreover, it has been reported that DHA can sensesize cisplatin to cisplatin-resistant ovarian cancer cells by inhibiting mTOR (Feng et al., 2014). DHA exerts anti-tumor effects in a multi-specific manner, in which it inhibits important tumor-related signaling pathways, such as Wnt/β-catenin, AMPK, PI3K/AKT, and so on (Efferth, 2017a). However, whether DHA could reverse cisplatin resistance in ESCC and its underlying mechanism need to be elucidated.

In this study, we demonstrated that Shh pathway was a key player of the cisplatin resistance in ESCC and DHA could inhibit Shh pathway to sensesize ESCC to cisplatin, suggesting that DHA may serve as a promising agent combined with cisplatin in ESCC treatment.

## Materials and Methods

### Cell Lines and Cell Culture

Human ESCC cell lines TE-1, EC109, and KYSE150 were obtained from Cell Bank of the Chinese Academy of Sciences (Shanghai, China). Human ESCC cell line KYSE510 was obtained from German Collection of Microorganisms and Cell Cultures (Germany). All cells were routinely cultured in RPMI-1640 or MEM medium supplemented with 10% fetal bovine serum (FBS) and maintained at 37°C in 5% CO_2_ and 100% humidity. For continuous treatment cells, the 1/2 and 1/4 IC_50_ concentrations of cisplatin were continuously given to cell lines for 2–3 weeks, and then cells were collected for next investigation.

### Patients and Tissue Specimens

A total of 59 ESCC patients who received platinum-based regimens were enrolled between Jan 2006 and Dec 2007 from Southwest Hospital (Chongqing, China). The enrolled patients met the following eligibility criteria: histological or cytological confirmation of esophageal squamous cell carcinoma, presence of measurable disease, no second malignancies, and availability of adequate diagnostic tumor tissues. The clinicopathologic features of these patients are summarized in Supplementary Table S1. Overall survival (OS) was defined as the time between the onset of chemotherapy and the date of the last follow up or death from any cause. Ethical oversight and approval were obtained from the Institutional Review Board of Southwest Hospital, and written informed consent was obtained from all patients.

### Immunohistochemistry

Immunohistochemical staining was performed on formalin-fixed, paraffin-embedded ESCC sections (4μm) using Dako REALTM EnVision^TM^ detection System (Code K5007; Dako, Glostrup, Denmark) as previously described (Liu et al., 2017). Briefly, the sections were pretreated by 0.3% H_2_O_2_ and antigen retrieval was performed according to the manufacturer’s instructions. The slides were incubated with mouse anti-human Shh (1:100; Cell Signaling Technology, United States) or Sox2 (1:100; Cell Signaling Technology, United States) at 4°C overnight. The secondary antibody (1:400; Jackson ImmunoResearch, United States) was added for incubation at 37°C for 30 min. All slides were evaluated independently by two pathologists in a blind manner. The intensity of immunohistochemical staining and the proportion of positively stained tumor cells was evaluated as previously described (Liu et al., 2017). Briefly, the staining intensity was scored with “0” (no staining), “1” (weakly positive), “2” (moderately positive), and “3” (strongly positive). The staining average percentage of positive cells was scored as: 0 = 0%, 1 = 1–25%, 2 = 26–50%, 3 = 51–75%, and 4 = 76–100%. The expression levels of detected proteins were reported by multiplication of staining density and average percentage of positive cells. It was defined as high expression if calculation of the scores more than 4 or 2 for Shh and Sox2, respectively, and the cut-off was derived from X-tile analysis, other tumor tissues were considered as low expression.

### Compounds and Reagents

Dihydroartemisinin (DHA) and cisplatin (CDDP) were obtained from Sigma, United States. These agents were dissolved in DMSO to 100 mM and stored at −20°C. Before treatment, the stock solution was diluted to different concentrations. The final concentration of DMSO in cultures was 0.1% (v/v) or less. MTT (3-(4,5-dimethylthiazol-2-yl)-2,5-diphenyl tetrazolium bromide) was purchased from Sigma, United States. The primary antibodies against Shh, PTCH1, Gli1, Nanog, Sox2, Oct4, P-gp, ALDH1A1, Tubulin, and β-actin were listed in Supplementary Table S2.

### Cell Viability Assay

The *in vitro* cell viability was determined by MTT assay. The cells (/mL) were seeded into 96-well culture plates at 5 × 10^3^ cells/well in 100 μL DMEM containing 10% FBS. After incubation overnight, the cells were treated with various concentrations of agents for 48 h. Then 10 μL MTT solution (2.5 mg/mL in PBS) was added to each well, and the plates were incubated for an additional 4 h at 37°C. After centrifugation (2,500 rpm, 10 min), the medium with MTT was aspirated, and replaced with 100 μL DMSO. The optical density of each well was measured at 570 nm with a Molecular Devices M5 Reader.

### Transient Transfection

For transiently silencing Shh, the Shh Silencer Select Validated siRNA was purchased from Life Technologies, United States (Catalog #AM16708). For overexpression of Shh, human Shh full-length cDNA was cloned into the pCMV expression vector. The Shh siRNA and pCMV-Shh (2 μg/μL) was transiently transfected into TE-1 or KYSE-510 cells by Lipofactamine 2000 (Invitrogen) according to the manufacturer’s instructions. Transfection efficiency was verified by western blotting.

### ALDEFLUOR Assay and Flow Cytometry

An ALDEFLUOR kit (Stem Cell Technologies, Canada) was used to isolate tumor cells with ALDH enzymatic activity according to the manufacturer’s instruction. Briefly, ESCC cells were suspended in ALDEFLUOR assay buffer containing ALDH substrate, then treated with BODIPY-aminoacetaldehyde (BAAA) and incubated for 40min at 37°C. Diethylaminobenzaldehyde (DEAB), a specific ALDH inhibitor, was used as a negative control. 7-Amino-actinomycin D (7-AAD) staining solution (BD Pharmingen, United States) was used to exclude dead cells. Cells with intact plasma membranes were sorted for experiments.

### Western Blot Analysis

ESCC cells or FACS-sorted ALDH^high^ cells were gathered after pre-treatment for the indicated time periods as described previously (Liu et al., 2017). Western blotting was performed as previously described (Wang et al., 2014). Briefly, equal amounts of total protein extracts from cultured cells or tissues were fractionated by 8–12% SDS-PAGE and then electrically transferred onto polyvinylidene difluoride (PVDF) membranes. Mouse or rabbit primary antibodies and appropriate horseradish peroxidase (HRP)-conjugated secondary antibodies were used to detect the designated proteins. The bound secondary antibodies on the PVDF membrane were reacted with ECL detection reagents (Pierce; Rockford, United States) and detected by a ChemiDocXRS system (Bio-Rad). β-actin or Tubulin were used as loading control.

### Quantitative RT-PCR Analysis

Total RNA was isolated from cells using RNeasy Mini Kits (Qiagen) as described in the product insert. The RNA was reverse transcribed with RevertAid First Strand cDNA Synthesis Kits (Thermo Fisher Scientific) and PCR was done using iQ SYBR Green Supermix and the CFX96 Real-Time PCR Detection System (Bio-Rad). Primers used in this study were listed in Supplementary Table S3. The expression of each gene was determined using the 2^–ΔΔCT^ method. Results were normalized against *GAPDH*. All experiments were performed in triplicate, and results were plotted as the mean ± s.d.

### Real-Time Cell Analysis (RTCA)

The RTCA assay was performed as previously described (Wang et al., 2015). A chemotactic signal for movement was provided by inoculating 30,000–50,000 TE-1 cells in serum-free medium in the upper chamber and supplying RPMI-1640 with 10% FBS in the lower chamber. Cell index (electrical impedance) was monitored every 5 min for the duration of the experiment. The cell index represents the capacity of cell migration, and the slope of the curve was related to the migration velocity of tumor cells.

### Colony Formation Assay

One hundred viable FACS-sorted ALDH^high^ TE-1 cells per well were seeded in each well of 24-well plates and cultured in DMEM containing 10% FBS. After incubation for 2 weeks, the colonies were stained with crystal violet and the colonies containing more than 50 cells were counted.

### Tumorsphere Formation Assay

FACS-sorted ALDH^high^ cells were seeded in 6-well ultra-low attachment plates (Corning, NY) at 500 cells/well with or without the indicated treatments in serum-free DMEM/F-12 medium supplemented with B27 (1×, Invitrogen), 20 ng/mL human recombinant bFGF (PeproTech), 20 ng/mL EGF (PeproTech), 10 ng/mL leukemia inhibitory factor (Chemicon) and 4 U/L insulin (Sigma). After culture for 2 weeks, spheres were counted under a phase contrast microscope, and pictures were taken by AF6000 and DFC350FX (Leica).

### Immunofluorescence Confocal Microscopy

FACS-sorted ALDH^high^ cells were seeded on cover slides and cultured in DMEM containing 10% FBS for 7 days with or without treatment. The cells were then fixed and blocked by preimmune goat serum. Primary mouse anti-human Gli1 (1:100, Cell Signaling, United States) was added to the cells and incubated at 4°C overnight. Secondary goat anti-mouse IgG conjugated with Cy5 (1:500, Cell Signaling, United States) was added to the cells and incubated at 37°C for 30 min. DAPI was used to stain the nuclei. Cells were then observed under laser confocal microscopy (Zeiss, Germany).

### Determination of Combination Index

TE-1 or KYSE-510 cells were treated with different concentrations of DHA alone, cisplatin alone, or the two agents in combination. The cell viability was measured by MTT assay. The nature of the drug interaction was analyzed by using the combination index (CI) according to the method of Chou and Talalay (1984). A CI value lower than 0.90 indicates synergism; a CI value between 0.90 and 1.10 indicates an additive effect; and a CI value higher than 1.10 indicates antagonism. Data analysis was performed using Calcusyn software (Biosoft, Oxford, United Kingdom).

### Mouse Xenograft Tumor Study

For tumorigenesis assessment, viable TE-1 cells (1 × 10^6^/100 μL PBS per mouse), as confirmed by trypan blue staining, were subcutaneously injected into the right flank of 7- to 8-week-old female BALB/c mice. When the average tumor volume reached 100 mm^3^, the mice were randomly divided into four treatment groups, including control (saline only, *n* = 4), DHA (25 mg/kg/5 days/week, i.p.; *n* = 4), CDDP (4 mg/kg/week, i.p.; *n* = 4), and the combination (*n* = 4). The dosage of CDDP and DHA was designed based on the clinical equivalent dose. Tumor size was measured once every 3 days with a caliper (calculated volume = shortest diameter^2^ × longest diameter/2). The body weight was also measured once every 3 days to assess gross toxicity. After 35 days, the mice were sacrificed and the tumors were excised and stored at −80°C until western blot analysis. These studies were performed in strict accordance with the recommendations in the Guide for the Care and Use of Laboratory Animals of the National Institutes of Health. The protocol was approved by the Committee on the Ethics of Animal Experiments of the Shenyang Pharmaceutical University.

### Statistical Analysis

Differences between experimental groups were evaluated by one-way ANOVA with Turkey’s *post-hoc* test using the SPSS11.5 software package for Windows (SPSS, Chicago, IL). Survival curves were constructed using the Kaplan–Meier method. Statistical significance was based on a *P*-value of 0.05 (*P* < 0.05, two-tailed test).

## Results

### Activation of the Shh Pathway Is Associated With Poor Outcome of ESCC Patients Receiving Platinum-Based Regimens

**FIGURE 1:**
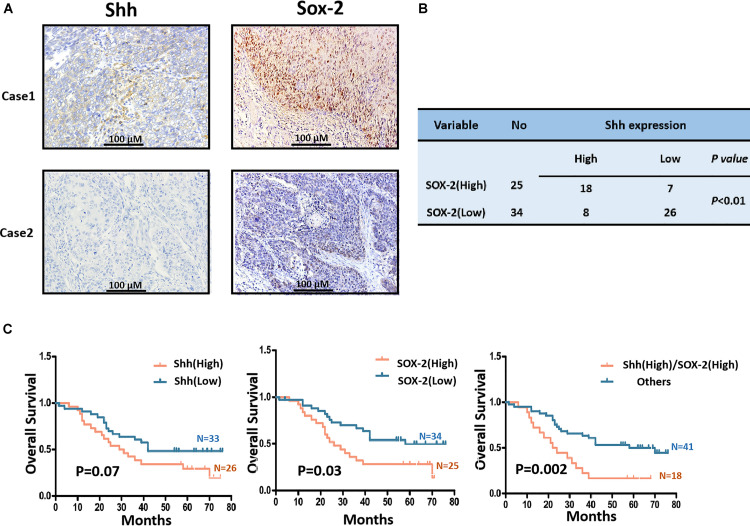
The expression of Shh and Sox2 in ESCC tissues derived from platinum-treated patients and its relationship with prognosis of the patients. **(A)** The representative images of immunohistochemistry staining for Shh and Sox2 proteins in ESCC tissues. **(B)** The correlation between Shh and Sox2 expression in ESCC. **(C)** Kaplan–Meier estimated overall survivals of platinum-treated ESCC patients according to expression levels of Shh or/and Sox2 in ESCC tissues.

In order to investigate whether the activation of Shh signaling was correlated to platinum resistance of ESCC, we first used immunohistochemistry (IHC) to measure the expression of Shh and Sox2, where Shh is a ligand and Sox2 is a target in canonical Shh signaling (Rimkus et al., 2016), in paraffin-embedded tumor tissues from 59 ESCC patients who were treated with platinum-based regimens. The Shh protein was mainly detected in the membrane and cytoplasm and Sox2 staining was mainly existed in nuclei in ESCC cells (Figure 1A). The cases with high expression of Shh and Sox2 were 26/59 (44.1%) and 25/59 (42.4%), respectively. The cases with both Shh and Sox2 high expression were 18/59 (30.5%). There was a positive relationship between Shh and Sox2 expression (*P* < 0.05, Figure 1B). Analysis of the relationship between expression of Shh and Sox2 and overall survival (OS) of the patients showed that the expression of Shh was marginally related to the OS of platinum-treated ESCC patients (*P* = 0.07, Figure 1C left), whereas Sox2 showed a significant correlation with the patients’ OS (*P* = 0.03, Figure 1C middle). Importantly, we found that both Shh and Sox2 high expression group was much more strongly correlated with poor prognosis than the “other” group included either single- or double-lower Shh and Sox2 (*P* = 0.002, Figure 1C right). Taken together, these results suggest that the activation of Shh signaling is involved in efficacy of platinum-based treatments in ESCC patients.

### The Activation of Shh Signaling Is Correlated With the Response of ESCC Cells to Cisplatin

**FIGURE 2:**
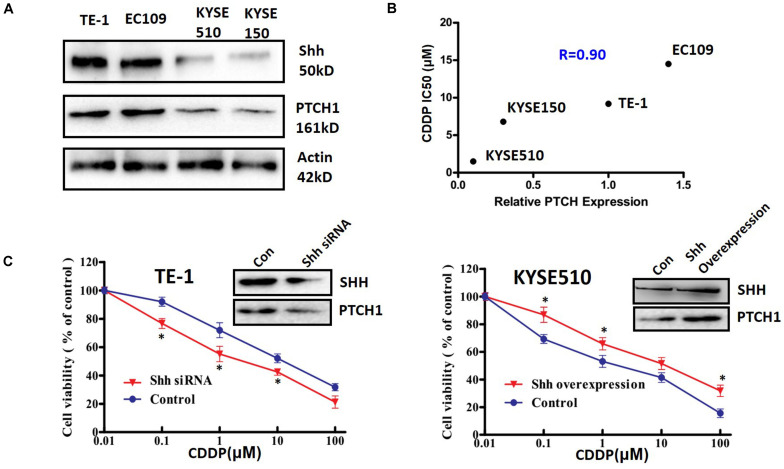
The relationship between the Shh signaling pathway and the sensitivity to cisplatin in ESCC cell lines. **(A)** The expression of Shh and PTCH1 in ESCC cell lines. **(B)** The correlation between PTCH expression level and sensitivity to cisplatin (CDDP) in ESCC cell lines. IC_50_ values were determined by the MTT method after treatment with CDDP for 48 h. **(C)** The effect of manipulating Shh expression on the cytotoxicity of CDDP in TE-1 and KYSE510 cells. The cells were transfected with specific siRNA in TE-1 cells (left panel) or pCMV-Shh vector in KYSE510 cells (right panel) for 48 h, and then treated with different concentrations of CDDP for another 48 h. All error bars are s.e.m. *P < 0.05, compared with control group.

To further confirm the correlation between Shh signaling and platinum sensitivity, we measured the expression levels of Shh and PTCH1, which is both a receptor and a downstream transcriptional target of Shh signaling, and the cytotoxic effect of cisplatin (CDDP) in four ESCC cell lines. TE-1 and EC109 cells expressed Shh and PTCH1 at a relatively higher levels than that in KYSE510 and KYSE150 cells (Figure 2A), suggesting that TE-1 and EC109 cells had a higher innate Shh signaling activity than that of KYSE510 and KYSE150 cells. In accordance with the expression levels of Shh and PTCH1, the IC_50_ values of TE-1 and EC109 cells on cisplatin were significantly higher than that of KYSE510 and KYSE150 cells, implying that the Shh signaling activity was negatively correlated with the sensitivity of ESCC cells to cisplatin (*R* = 0.9; Figure 2B). These results were confirmed by manipulating the expression of Shh in ESCC cells. Transient transfection with siRNA targeted Shh markedly reduced the expression of Shh and PTCH1 (Figure 2C left upper panel) and significantly increased cisplatin-induced cytotoxicity (Figure 2C left lower panel) in TE-1 cells. Inversely, overexpression of Shh in KYSE510 cells resulted in a significant upregulation of PTCH1 expression (Figure 2C right upper panel) and reduction of cisplatin-induced cytotoxicity (Figure 2C right lower panel). These results suggest that the Shh pathway plays a key role in the sensitivity of ESCC cells to cisplatin.

### Continuous Cisplatin Treatment Activates Shh Signaling and Induces Cancer Stem-Like Properties in ESCC Cells

**FIGURE 3:**
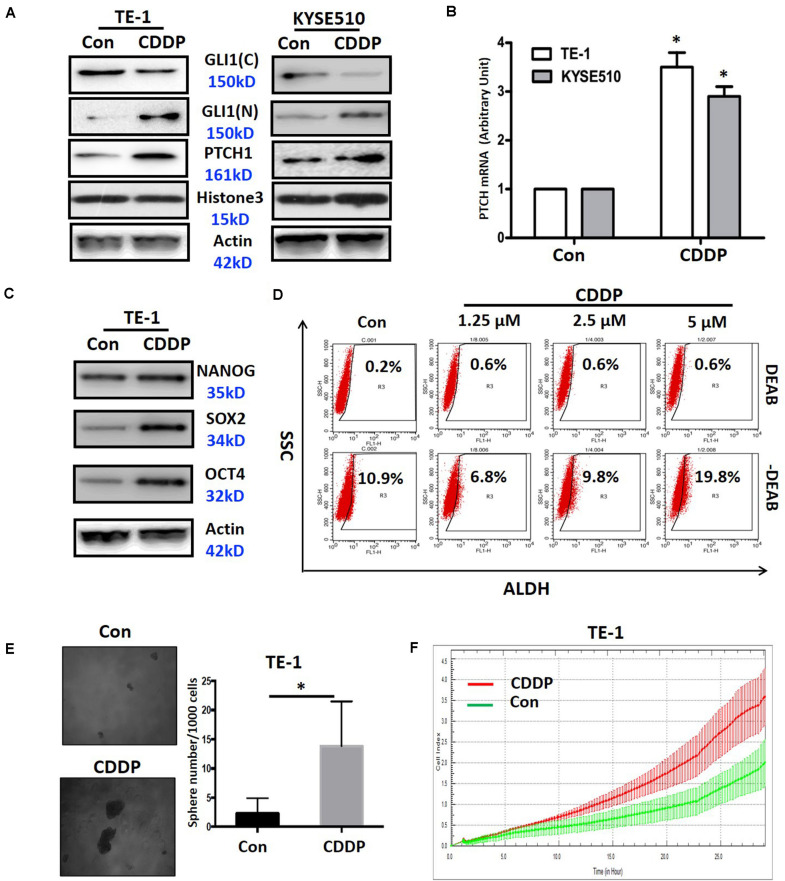
The effects of cisplatin on activation of the Shh pathway and related biological functions. **(A)** The protein expression of Gli1 (nuclear and cytoplasmic) and PTCH1 in TE-1 and KYSE510 cells continuously treated with cisplatin (CDDP). Histone3 and β-actin expression were used as nuclear and total loading controls, respectively. **(B)** The mRNA expression of PTCH1 in TE-1 and KYSE510 cells continuously treated with CDDP. *GAPDH* expression was used as a control. *P < 0.05, compared with control group cells. **(C)** The expression of the cancer stem cell-related transcription factors Nanog, Sox2, and Oct4 in TE-1 cells after continuous exposure to CDDP. β-actin expression was used as a loading control. **(D)** The percentages of ALDH1 positive cells in TE-1 cells after continuous exposure to CDDP. **(E)** Sphere formation after continuous exposure to CDDP in TE-1 cells. *P < 0.05, compared with control group. **(F)** The effect of continuous exposure to CDDP on cell migration in TE-1 cells.

To further clarify the linkage of cisplatin resistance to the activation of Shh signaling pathway, we continuously treated ESCC cells with cisplatin to observe the activation status of Shh pathway in TE-1 and KYSE510 cell lines. As shown in Figure 3A, continuous cisplatin treatment resulted in a significant nuclear translocation of Gli1 in TE-1 and KYSE510 cells, which represented the activation of Shh signaling. Consistent with the activation of the Shh pathway, the expression of the downstream transcriptional target PTCH1 was markedly increased at the mRNA and protein levels in both cell lines (Figures 3A,B). It is well known that Shh signaling is involved in the maintenance of cancer stem cell (CSC) traits, such as self-renewal and drug resistance (Ruiz i Altaba et al., 2002). Thus, we next observed the effect of continuous treatment of cisplatin on CSC properties. The results showed that continuous cisplatin treatment induced the upregulation of CSC related transcription factors, including Nanog, Sox2, and Oct4 in TE-1 cells (Figure 3C) and increased the proportion of ALDH1 positive cells, i.e., ESCC stem cells (Yang et al., 2014; Figure 3D). Cisplatin-treated TE-1 cells also showed enhanced tumor sphere formation (Figure 3E) and increased migration capability (Figure 3F). These results indicate that continuous exposure of ESCC cells to cisplatin lead to the activation of Shh signaling and the accumulation of ESCC stem cells.

### Dihydroartemisinin Suppresses the Activation of Shh Pathway and Attenuates the Cancer Stem-Like Traits in ESCC Cells

**FIGURE 4:**
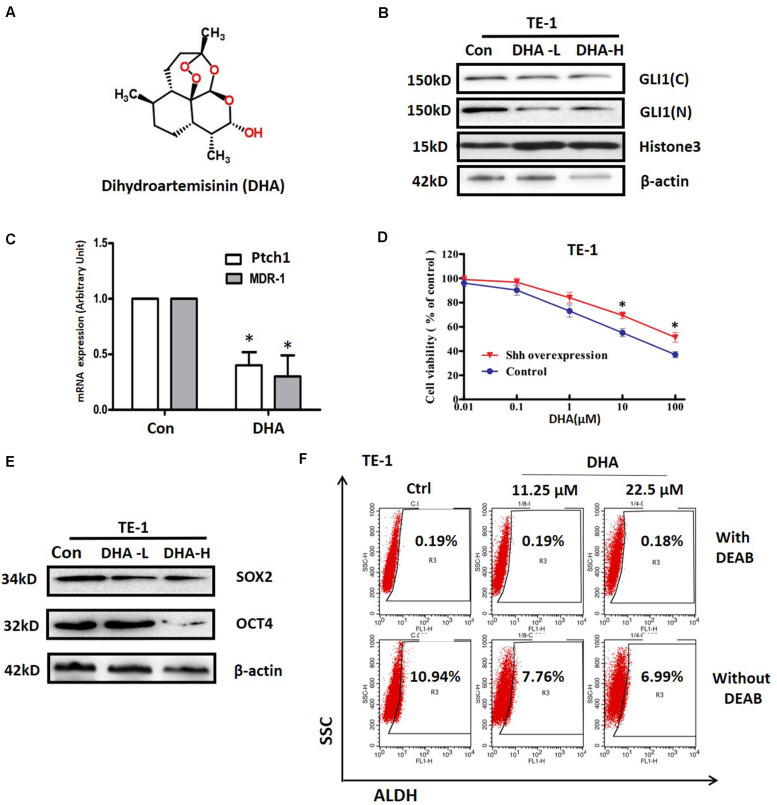
The effects of DHA on activation of Shh pathway and stemness in ESCC cells. **(A)** The structure of DHA. **(B)** The effect of DHA at 2 μM (low concentration, DHA-L) and 20 μM (high concentration, DHA-H) on expression of Gli1 in the nucleus and cytoplasm in TE-1 cells. Histone3 and β-actin expression were used as nuclear and total loading controls, respectively. **(C)** The effect of DHA on the expressions of *PTCH* and *MDR1* at mRNA level in TE-1 cells. *P < 0.05, compared with DMSO treated group cells. **(D)** Effect of DHA on the viability of TE-1 cells with or without Shh overexpression vector transfection. The cells were transfected with Shh overexpression or mock vector (control) for 48 h, and then treated with different concentrations of DHA for another 48 h. **(E)** The effects of DHA on the expression of Sox2 and Oct4 proteins in TE-1 cells. β-actin expression was used as a loading control. **(F)** The effect of DHA on the proportion of ALDH1 positive cells in TE-1 cells. All error bars are s.e.m. *P < 0.05, compared with control group.

Dihydroartemisinin (DHA, Figure 4A), a sesquiterpene lactone isolated from the traditional Chinese herb *Artemisia annua*, was reported to have antitumorigenic properties and has been tested in clinical trials as a new agent to treat various cancers (Odaka et al., 2014; Lucibello et al., 2015; Qin et al., 2015). We treated TE-1 cells with DHA and found that it significantly inhibited nuclear translocation of Gli1 (Figure 4B) and downregulated the transcription of the Shh target genes *PTCH1* and *MDR1* (Figure 4C). These results suggest that DHA might be a novel inhibitor of the Shh signaling pathway in ESCC cells. So we further observed whether overexpressing Shh would affect the roles of DHA in TE-1 cells. As shown in Figure 4D, overexpression of Shh significantly attenuated the DHA-induced cytotoxicity (*P* < 0.05), implying that the Shh signaling pathway was an important target of DHA in ESCC cells. Since Shh signaling is a classical cancer stem cell-related pathway, so we next examined whether DHA could affect the property of cancer stem cells. As shown in Figure 4E, DHA decreased the expression level of the stem cell-related transcription factors Sox2 and Oct4 in TE-1 cells. Furthermore, flow cytometry assay data indicated that DHA treatment also decreased the proportion of ALDH1 positive cells in TE-1 cells (Figure 4F). These results identify DHA as a novel inhibitor of the Shh pathway with the ability to suppress the cancer stem-like properties in ESCC cells.

### The Combination of Dihydroartemisinin and Cisplatin Synergistically Suppress the Cell Proliferation and the Stem Cell Properties in ESCC Cells

**FIGURE 5:**
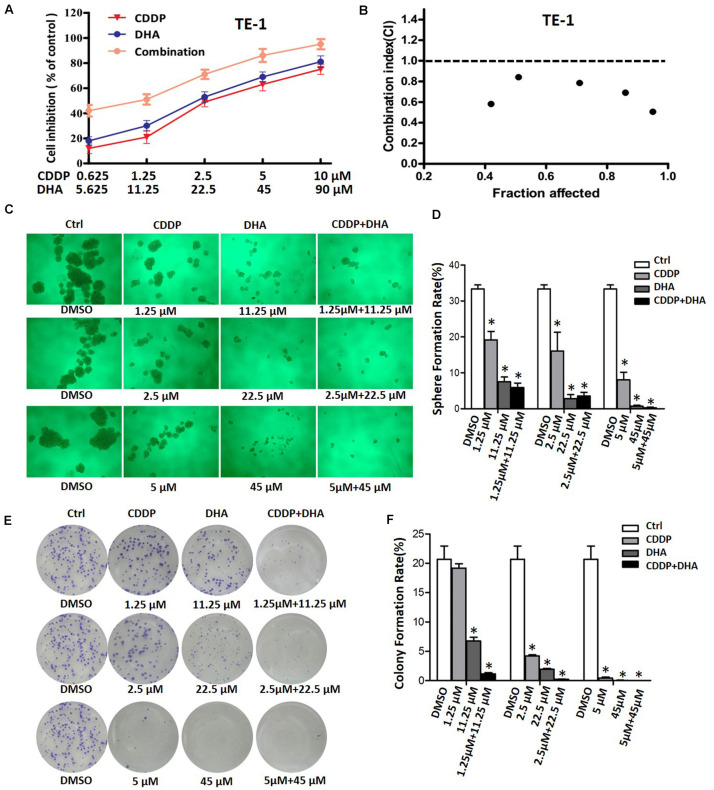
The effects of DHA, cisplatin, and their combination on cell viability and sphere and colony formation. **(A)** Growth curves of TE-1 cells after treatment with DHA, cisplatin (CDDP), and their combination. **(B)** Analysis of the combination of DHA and CDDP in TE-1 cells. The cells were treated by using various concentrations of DHA and CDDP, either alone or in a fixed ratio for 48 h. The combination index was analyzed using the Calcusyn program. **(C)** Representative images for sphere formation of ALDH + TE-1 cells after treatment with DHA, CDDP, and DHA + CDDP at designated concentrations for 24 h. **(D)** Bar graph of sphere formation efficiency of ALDH + TE-1 cells after treatment with DHA or/and CDDP. *P < 0.05, compared with DMSO treated group. **(E)** Representative images of colony formation of ALDH + TE-1 cells after treatment with DHA, CDDP and DHA + CDDP at designated concentrations for 24 h. **(F)** Bar graph of colony formation efficiency of ALDH + TE-1 cells after treatment with DHA or/and CDDP. *P < 0.05, compared with DMSO treated group.

We next assessed the growth inhibitory effect of DHA in combination with cisplatin on ESCC cells. In TE-1 cells, which have high innate Shh signaling activity, the combination of DHA and cisplatin suppressed proliferation more effectively than DHA or cisplatin alone (Figure 5A). Combination analysis showed that DHA/cisplatin together led to a strong synergistic antiproliferative effect on TE-1 cells with a minimal combination index (CI) of 0.506 (Figure 5B). However, in KYSE-510 cells, which have lower innate Shh activity, the combination of DHA and cisplatin did not have a synergistic antiproliferative effect (minimal *CI* = 1.411; see Supplementary Figure S1). The above results demonstrate that the synergistic effect of DHA and cisplatin is associated with the Shh signaling activity in ESCC cells.

In view of the relationship between Shh signaling and the cancer stem cell property, we further verified the synergistic effect of DHA and cisplatin on ESCC stem cells. As shown in Figures 5C–F, cisplatin alone inhibited sphere and colony formation of ALDH + TE-1 cells. In comparison to cisplatin, DHA at equal effective concentrations (calculated by IC_50_) displayed an enhanced inhibitory effect. Importantly, the combination of DHA and cisplatin had an obvious synergistic anti-self renewal effect on ALDH + TE-1 cells, that is which exhibited stronger inhibitory effect on sphere and colony formation than DHA and cisplatin alone (Figures 5E,F). These results indicated that DHA and cisplatin synergistically inhibit the proliferation of bulk cells and the self-renewal of cancer stem cells in ESCC cells.

To further explore the effect of the cisplatin/DHA combination on ESCC stem cells, we analyzed the levels of cancer stem cell markers. The results showed that cisplatin alone upregulated the expression of Sox2, Oct4, and ALDH1A1 at the mRNA and protein levels. The combination of DHA and cisplatin inhibited the upregulation of Sox2, Oct4, and ALDH1A1 induced by continuous cisplatin treatment (see Supplementary Figures S2A,B). Taken together, our results indicate that the combination of DHA and cisplatin synergistically suppresses the ESCC stem cell properties.

### DHA Enhances Cellular Enrichment of Cisplatin and Blocks Cisplatin-Induced Activation of Shh Signaling in TE-1 Cells

**FIGURE 6:**
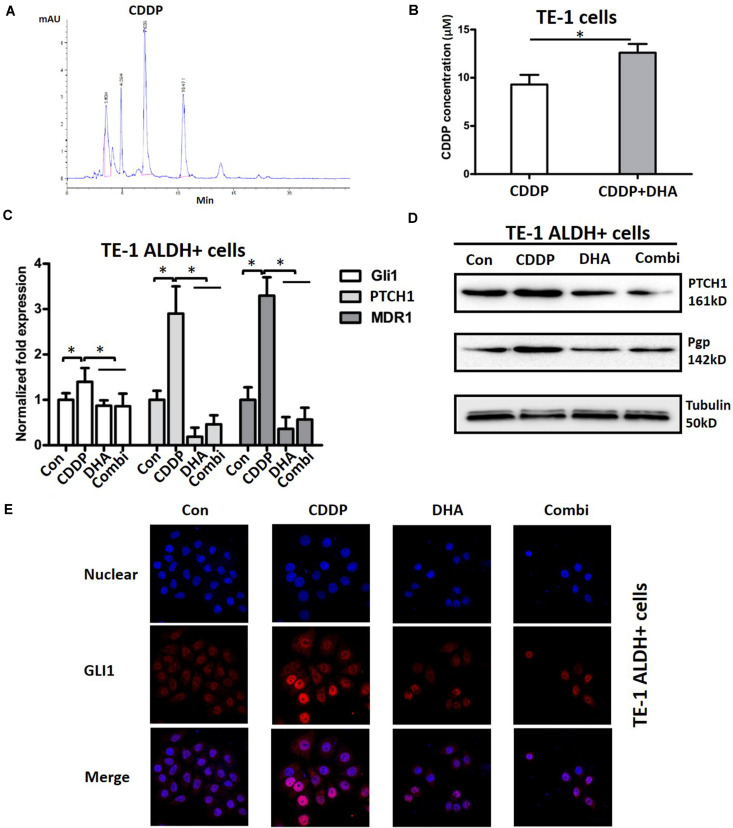
The effects of DHA on the cellular cisplatin content and cisplatin-induced activation of the Shh pathway. **(A)** Representative HPLC trace showing the cisplatin (CDDP) peak. **(B)** Analysis of the cellular concentration of CDDP in TE-1 cells with or without DHA. **(C)** The expression of *Gli1*, *PTCH1*, and *MDR1* mRNAs in ALDH + TE-1 cells after treatment with DHA (11.25 μM) or/and CDDP (1.25 μM) for 24 h. ^∗^*P* < 0.05, compared with CDDP treated group. **(D)** The expression of PTCH1 and P-gp proteins in ALDH + TE-1 cells after treatment with DHA (11.25 μM) or/and CDDP (1.25 μM) for 24 h. Tubulin expression was used as a loading control. **(E)** The effect of DHA or/and CDDP on Gli1 nuclear translocation in ALDH + TE-1 cells. ^∗^*P* < 0.05 for DHA plus CDDP compared with the DMSO plus CDDP.

The reduction of drug accumulation in the cells is a common mechanism for anticancer drugs (Fletcher et al., 2016). Considering the synergistic action of DHA in combination with cisplatin, we next used HPLC to examine whether DHA could enhance the enrichment of cisplatin in TE-1 cells. As shown in Figure 6A, the cisplatin in cell culture medium was detected by HPLC as a clear peak. Incubation of TE-1 cells with DHA resulted in a 35.4% increase of cellular cisplatin (Figure 6B), which suggests that DHA promotes cellular enrichment of cisplatin. In order to further investigate the mechanisms underlying the synergistic action, we investigated the effect of cisplatin alone, DHA alone, or the cisplatin/DHA combination on Shh signaling. As shown in Figure 6C, cisplatin single treatment upregulated the mRNA levels of the Shh signaling target genes *Gli1*, *PTCH1*, and *MDR1*, a gene that promotes cisplatin efflux in ALDH + TE-1 cells, whereas these effects were completely blocked by DHA (Figure 6C). A similar pattern was also observed at the protein level. DHA in combination with cisplatin caused an obvious downregulation of PTCH1 and Pgp as compared to cisplatin single treatment (Figure 6D). We also assessed the nuclear location of Gli1 by using confocal microscopy. As shown in Figure 6E, cisplatin treatment led to the accumulation of Gli1 in nuclei, and this co-treatment with DHA blocked the nuclear translocation of Gli1 in TE-1 cells. The above data demonstrate that DHA synergistically enhances the anti-tumor effect of cisplatin in ESCC cells by blocking cisplatin-induced activation of Shh signaling.

### DHA Combined With Cisplatin Synergistically Retards Tumor Growth *in vivo* by Inhibiting Shh Signaling

**FIGURE 7:**
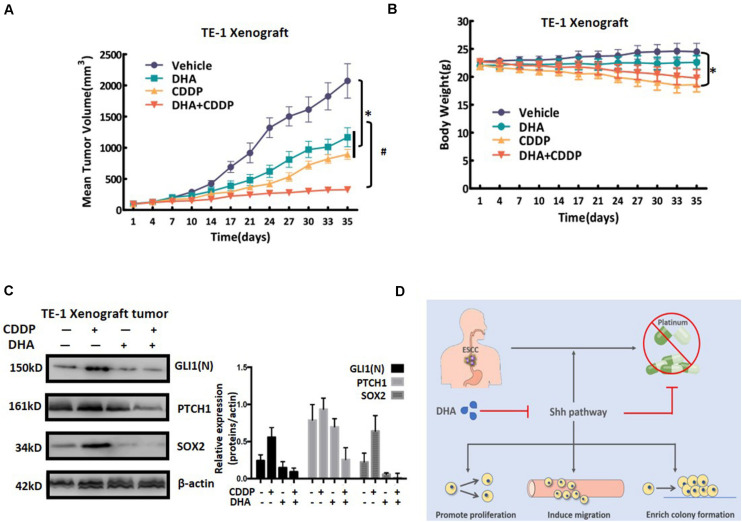
The anti-tumor effect of DHA and cisplatin on TE-1-derived xenografts in mice. **(A)** The effect of administration of DHA alone, cisplatin (CDDP) alone or the DHA/CDDP combination on the tumor volumes. Tumor volumes are expressed as mean ± SD (*n* = 4 per group). **(B)** The average body weight of each group is expressed as mean ± SD (*n* = 4 per group). **(C)** The expression levels of nuclear Gli1, PTCH1, and Sox2 were detected by western blot. Proteins were extracted from the tumor tissues of mice in the four groups. The expression levels of nuclear Gli1, PTCH1, and Sox2 were detected by western blot. Proteins were extracted from the tumor tissues of mice in the four groups. **(D)** Schematic of the mechanism by which Shh pathway activation results in cisplatin resistance in ESCCs and reversing resistance by novel Shh pathway inhibitor DHA. All error bars are s.e.m. ^∗^*P* < 0.05 for DHA alone, CDDP alone or the combination treatment compared with the vehicle control. ^#^*P* < 0.05 for the combination treatment compared with single administration of DHA and CDDP.

Next we assessed the effects of the combination strategy of DHA and cisplatin *in vivo*. BALB/c nude mice bearing TE-1 xenografts were treated with cisplatin, DHA, or cisplatin plus DHA. As shown in Figure 7A, cisplatin or DHA single administration resulted in a moderate inhibition of tumor growth, with inhibition rates of 56.8 and 43.6%, respectively. Compared to the single treatments, cisplatin plus DHA treatment displayed a much more obvious inhibition of tumor growth (inhibition rate 84.2%), which confirmed our *in vitro* results. Additionally, the gross toxicity data showed that cisplatin group had a significant decrease in the body weights of the mice, whereas DHA group had not, as compared to control group. Importantly, the body weights of mice treated with the combination of cisplatin and DHA showed a weak increase when compared with cisplatin alone (Figure 7B). The effects of DHA, cisplatin and the combination of cisplatin and DHA on Shh signaling was confirmed by testing the expression levels of nuclear Gli1, PTCH1, and Sox2 in tumor cells obtained from xenografts (Figure 7C). Xenografts treated with cisplatin alone exhibited increased expression levels of nuclear Gli1, PTCH1, and Sox2, which suggests that Shh signaling was activated. However, treatment with the combination of cisplatin and DHA led to an inhibition of Shh activity. These results indicate that DHA combined with cisplatin synergistically retarded tumor growth by overcoming cisplatin-induced activation of Shh signaling *in vivo*.

## Discussion

Platinum derivatives, the best known of which is cisplatin, are currently employed as a key component in the first-line standard of care for the clinical treatment of patients with ESCC (Nakajima and Kato, 2013; Rubenstein and Shaheen, 2015). Despite the unresolved issues regarding its mechanism of action, treatment with platinum-based drugs is generally associated with high rates of clinical response. However, accumulating evidence now suggests that malignant cells continuously exposed to cisplatin will activate adaptive responses that render them less susceptible to the antiproliferative and cytotoxic effects of the drug, and will eventually resume proliferation (Galluzzi et al., 2014). Therefore, the vast majority of cisplatin-treated ESCC patients are destined to experience the emergence of acquired cisplatin resistance and tumor recurrence.

Delineation of the molecular mechanisms whereby ESCC cells progressively lose their sensitivity to cisplatin will pave the way for rationally designed and hopefully long-lasting combinations of targeted therapies. In the present study, we found that the Shh signaling activity was increased in the tumors of platinum-treated ESCC patients with poor survival. Furthermore, the activation of Shh signaling was negatively correlated with the sensitivity of ESCC cell lines to cisplatin. Interestingly, continuous treatment of ESCC cells with cisplatin also resulted in the activation of Shh signaling along with the enhanced cancer stem cell property. Importantly, our results indicate that inhibition of Shh signaling by DHA could be a strategy to overcome acquired resistance to cisplatin. Overall, our results have important implications for clinical oncology and ESCC biology.

Feedback activation of oncogenic pathways is considered as an important mechanism to mediate acquired drug resistance in tumors. Recently, several studies have shown that feedback activation of oncogenic pathways is also involved in the cisplatin resistance in ESCC. Myers et al. (2015) reported that IGF signaling, together with the MAPK or PI3K/AKT signaling pathways, was activated in cisplatin-resistant ESCC patients, and inhibition of the IGF or/and MAPK and PI3K/AKT signaling pathways could sensitize ESCC cells to cisplatin. Based on clinical pathological and *in vitro* data, Sugimura et al. (2012) demonstrated that the interleukin-6/STAT3 prosurvival pathway was activated in ESCC cells by cisplatin treatment, and inhibition of that feedback pathway by let-7c restored sensitivity to cisplatin. Here, we found for the first time that the Shh signaling pathway was activated in cells continuously exposed to cisplatin compared to the parental cells, and this activation resulted in an enhanced migratory capability and cancer stem cell characteristics, which suggests a novel biological role of the Shh signaling pathway in cisplatin resistance. Furthermore, the relationship between Shh activity and outcome in cisplatin-treated ESCC patients implies that Shh pathway activity might serve as a predictive biomarker for cisplatin treatment.

Besides feedback activation of oncogenic pathways, the classical mechanisms of cisplatin resistance are widely recognized to include increased DNA repair, altered cellular accumulation of drug and increased drug inactivation (Amable, 2016). Here, we found that the Shh signaling pathway is involved in cisplatin resistance. Two lines of evidence suggest how Shh signaling may be linked to the classical resistance mechanisms. First, MDR1 is considered to be involved in the process of altered cellular cisplatin accumulation and increased cisplatin inactivation (Sims-Mourtada et al., 2007), and *MDR1* is also recognized as a target gene of the Shh signaling pathway (Kudo et al., 2012). Consistent with previous reports, our results showed that inhibition of *MDR1* expression by DHA enhanced the enrichment of cellular cisplatin. Secondly, Kudo and his co-workers demonstrated that inhibition of the Shh pathway by shRNA led to the downregulation of three genes essential to nucleotide excision repair (*ERCC1*, *XPD*) and base excision repair (*XRCC1*) (Kudo et al., 2012), which mediated cisplatin resistance by increasing DNA repair. These published results indicate that the Shh signaling pathway might also be involved in other cisplatin resistance mechanisms.

The essential role of the Shh signaling pathway in ESCC cisplatin resistance in our studies and the limitation of an FDA-approved Shh inhibitor prompted us to identify a novel Shh inhibitor. In the current study, we identified DHA as a novel inhibitor of the Shh signaling pathway. Our results indicated that DHA inhibits the nuclear translocation of Gli1, one of the key transcription factors in the Shh signaling pathway, and suppresses the transcription of *PTCH* and *MDR1* which are target genes of the Shh pathway. Furthermore, overexpression of Shh ligand resulted in decreased sensitivity of ESCC cells to DHA. The above results demonstrate the inhibitory role of DHA in the Shh signaling pathway. Consistent with the Shh activity data, our functional studies also indicated that DHA reduced the expression level of cancer stem cell transcription factors and inhibited ALDH activity, which is recognized as an ESCC stem cell marker. However, the detailed mechanism by which DHA inhibits the Shh signaling pathway needs to be further elucidated.

Although the cisplatin resistance mechanisms in ESCC have been a subject of considerable interest, the development of therapeutic strategies that reverse cisplatin resistance has remained a challenge in clinical oncology. The cisplatin-induced increase of Shh activity and the inhibitory action of DHA on the Shh pathway prompted us to evaluate a combination strategy to overcome cisplatin resistance *in vitro* and *in vivo*. Our results showed that DHA in combination with cisplatin displayed a synergistic inhibitory effect *in vitro* and *in vivo* on TE-1 ESCC cells, which have relatively high Shh activity. The combination also synergistically inhibited the characteristics of ESCC stem cells. Given the limited capacity of cisplatin to control ESCC and the important role of the Shh pathway in the cisplatin resistance of patients, this work lays the foundation for a promising therapeutic strategy.

It should be noted that several clinical studies have demonstrated the combination of DHA or its derivatives and chemotherapy agents could obtained a promising efficacy in various tumor patients (Efferth, 2017b). The advantage of these combinations might be explained by the following fact: (1) DHA owns a known safety profile and pharmacokinetic characteristics (Efferth, 2017b); (2) Multiple action to suppress drug-resistant signaling pathways, such as Wnt, Shh, Stat3 and NF-κB pathways (Efferth, 2017b; Chaib et al., 2019). However, a recent study indicated that Artesunate, a DHA derivatives, could induce hepatoxicity when combined with temozolomide in the treatment for glioblastoma (Efferth, 2017b). Thus, optimizing dose should be addressed when combined chemotherapy agents with DHA in treatment for cancer.

## Conclusion

In conclusion, we provide evidence that activation of the Shh pathway is related to cisplatin resistance in ESCC and also to predicted poor overall survival of ESCC patients treated with cisplatin. Combining DHA with cisplatin suppressed the Shh signaling pathway, and thus synergistically inhibited cell growth and the characteristics of cancer stem cells *in vitro* and *in vivo* (see Figure 7D; Cui et al., 2020). Our findings not only elucidate a novel mechanism for cisplatin resistance, but also provide a promising therapeutic strategy to overcome cisplatin resistance in ESCC.

## Data Availability Statement

All datasets generated for this study are included in the article/Supplementary Material, further inquiries can be directed to the corresponding authors.

## Ethics Statement

The studies involving human participants were reviewed and approved by the Institutional Review Board of Southwest Hospital. The patients/participants provided their written informed consent to participate in this study. The animal study was reviewed and approved by Committee on the Ethics of Animal Experiments of the Shenyang Pharmaceutical University.

## Author Contributions

WC, XC, and LY: conceptualization, writing—original draft preparation, and writing—review and editing. TF, ZD, and DX: methodology. YW and MZ: validation. TF: formal analysis. TF, ZD, DX, and XC: investigation. TF and ZD: data curation. WC: supervision and project administration. WC and LY: funding acquisition. All authors have read and agreed to the published version of the manuscript.

## Conflict of Interest

The authors declare that the research was conducted in the absence of any commercial or financial relationships that could be construed as a potential conflict of interest.

## Supplementary Material

Supplementary Figure 1 The effect of CDDP, DHA, and combination on cell growth of KYSE-510 cells.

Supplementary Figure 2 The effect of CDDP, DHA, and combined treatment on the expressions of Sox-2, Oct4, and ALDH1A1 in TE-1 ALDH + cells.

Supplementary Table 1 The clinicopathological parameters of ESCC patients.

Supplementary Table 2 The information for antibodies used in this study.

Supplementary Table 3 The primer sequences for qRT-PCR analyses.

## References

[B1] Aichler M. Motschmann M. Jütting U. Luber B. Becker K. Ott K. (2014). Epidermal growth factor receptor (EGFR) is an independent adverse prognostic factor in esophageal adenocarcinoma patients treated with cisplatin-based neoadjuvant chemotherapy. *Oncotarget* 5 6620–6632. 10.18632/oncotarget.2268 25216514 PMC4196151

[B2] Amable L. (2016). Cisplatin resistance and opportunities for precision medicine. *Pharmacol. Res.* 106 27–36. 10.1016/j.phrs.2016.01.001 26804248

[B3] Arnold M. Soerjomataram I. Ferlay J. Forman D. (2015). Global incidence of oesophageal cancer by histological subtype in 2012. *Gut* 64 381–387. 10.1136/gutjnl-2014-308124 25320104

[B4] Athar M. Li C. Kim A. L. Spiegelman V. S. Bickers D. R. (2014). Sonic hedgehog signaling in basal cell nevus syndrome. *Cancer Res.* 74 4967–4975. 10.1158/0008-5472.can-14-1666 25172843 PMC4167483

[B5] Bain G. H. Collie-Duguid E. Murray G. I. Gilbert F. J. Denison A. McKiddie F. (2014). Tumour expression of leptin is associated with chemotherapy resistance and therapy-independent prognosis in gastro-oesophageal adenocarcinomas. *Br. J. Cancer* 110 1525–1534. 10.1038/bjc.2014.45 24569475 PMC3960617

[B6] Berman D. M. Karhadkar S. S. Maitra A. Montes, De Oca R. Gerstenblith M. R. (2003). Widespread requirement for Hedgehog ligand stimulation in growth of digestive tract tumours. *Nature* 425 846–851. 10.1038/nature01972 14520411

[B7] Cao B. Shi Q. Wang W. (2015). Higher expression of SIRT1 induced resistance of esophageal squamous cell carcinoma cells to cisplatin. *J. Thorac. Dis.* 7 711–719. 25973238 10.3978/j.issn.2072-1439.2015.04.01 PMC4419326

[B8] Chaib I. Cai X. Llige D. Santarpia M. Jantus-Lewintre E. Filipska M. (2019). Osimertinib and dihydroartemisinin: a novel drug combination targeting head and neck squamous cell carcinoma. *Ann. Transl. Med.* 7:651. 10.21037/atm.2019.10.80 31930052 PMC6944621

[B9] Chase D. M. Mathur N. Tewari K. S. (2010). Drug discovery in ovarian cancer. *Recent Pat Anticancer Drug Discov.* 5 251–260. 10.2174/157489210791760472 20524931

[B10] Chou T. C. Talalay P. (1984). Quantitative analysis of dose-effect relationships: the combined effects of multiple drugs or enzyme inhibitors. *Adv. Enzyme Regul.* 22 27–55. 10.1016/0065-2571(84)90007-4 6382953

[B11] Cui W. Wang L. H. Wen Y. Y. Song M. Li B. L. Chen X. L. (2010). Expression and regulation mechanisms of Sonic Hedgehog in breast cancer. *Cancer Sci.* 101 927–933. 10.1111/j.1349-7006.2010.01495.x 20180807 PMC11158104

[B12] Cui W. Fang T. Duan Z. Xiang D. Wang Y. Zhang M. (2020). Dihydroartemisinin sensitizes esophageal squamous cell carcinoma to cisplatin by inhibiting Sonic Hedgehog signaling. *Res. Square.* 10.3389/fcell.2020.596788 PMC7758349 33363149

[B13] Duan Z. H. Wang H. C. Zhao D. M. Ji X. X. Song M. Yang X. J. (2015). Cooperatively transcriptional and epigenetic regulation of sonic hedgehog overexpression drives malignant potential of breast cancer. *Cancer Sci.* 106 1084–1091. 10.1111/cas.12697 25990213 PMC4556399

[B14] Efferth T. (2017a). From ancient herb to modern drug: artemisia annua and artemisinin for cancer therapy. *Semin Cancer Biol.* 46 65–83. 10.1016/j.semcancer.2017.02.009 28254675

[B15] Efferth T. Schöttler U. Krishna S. Schmiedek P. Wenz F. Giordano F. A. (2017b). Hepatotoxicity by combination treatment of temozolomide, artesunate and Chinese herbs in a glioblastoma multiforme patient: case report review of the literature. *Arch. Toxicol.* 91 1833–1846. 10.1007/s00204-016-1810-z 27519711

[B16] Feng X. Li L. Jiang H. Jiang K. Jin Y. Zheng J. (2014). Dihydroartemisinin potentiates the anticancer effect of cisplatin via mTOR inhibition in cisplatin-resistant ovarian cancer cells: involvement of apoptosis and autophagy. *Biochem. Biophys. Res. Commun.* 444 376–381. 10.1016/j.bbrc.2014.01.053 24462866

[B17] Fletcher J. I. Williams R. T. Henderson M. J. Norris M. D. Haber M. (2016). ABC transporters as mediators of drug resistance and contributors to cancer cell biology. *Drug Resist Updat.* 26 1–9. 10.1016/j.drup.2016.03.001 27180306

[B18] Galanski M. (2006). Recent developments in the field of anticancer platinum complexes. *Recent Pat Anticancer Drug Discov.* 1 285–295. 10.2174/157489206777442287 18221042

[B19] Galluzzi L. Vitale I. Michels J. Brenner C. Szabadkai G. Harel-Bellan A. (2014). Systems biology of cisplatin resistance: past, present and future. *Cell Death Dis.* 5:e1257. 10.1038/cddis.2013.428 24874729 PMC4047912

[B20] Kudo K. Gavin E. Das S. Amable L. Shevde L. A. Reed E. (2012). Inhibition of Gli1 results in altered c-Jun activation, inhibition of cisplatin-induced upregulation of ERCC1, XPD and XRCC1, and inhibition of platinum-DNA adduct repair. *Oncogene* 31 4718–4724. 10.1038/onc.2011.610 22266871

[B21] Lai K. K. Chan K. T. Choi M. Y. Wang H. K. Fung E. Y. Lam H. Y. (2016). 14-3-3σ confers cisplatin resistance in esophageal squamous cell carcinoma cells via regulating DNA repair molecules. *Tumour Biol.* 37 2127–2136. 10.1007/s13277-015-4018-6 26346170

[B22] Li B. Tsao S. W. Chan K. W. Ludwig D. L. Novosyadlyy R. Li Y. Y. (2014). Id1-induced IGF-II and its autocrine/endocrine promotion of esophageal cancer progression and chemoresistance–implications for IGF-II and IGF-IR-targeted therapy. *Clin. Cancer Res.* 20 2651–2662. 10.1158/1078-0432.ccr-13-2735 24599933

[B23] Liu Q. Cui X. Yu X. Bian B. S. Qian F. Hu X. G. (2017). Cripto-1 acts as a functional marker of cancer stem-like cells and predicts prognosis of the patients in esophageal squamous cell carcinoma. *Mol. Cancer* 16:81. 10.1186/s12943-017-0650-7 PMC5399850 28431580

[B24] Lucibello M. Adanti S. Antelmi E. Dezi D. Ciafrè S. Carcangiu M. L. (2015). Phospho-TCTP as a therapeutic target of Dihydroartemisinin for aggressive breast cancer cells. *Oncotarget* 6 5275–5291. 10.18632/oncotarget.2971 25779659 PMC4467148

[B25] Luo H. Vong C. T. Chen H. Gao Y. Lyu P. Qiu L. (2019). Naturally occurring anti-cancer compounds: shining from Chinese herbal medicine. *Chin. Med.* 14:48. 10.1186/s13020-019-0270-9 PMC6836491 31719837

[B26] Merchant J. L. (2012). Hedgehog signalling in gut development, physiology and cancer. *J. Physiol.* 590 421–432. 10.1113/jphysiol.2011.220681 22144577 PMC3379690

[B27] Myers A. L. Lin L. Nancarrow D. J. Wang Z. Ferrer-Torres D. Thomas D. G. (2015). IGFBP2 modulates the chemoresistant phenotype in esophageal adenocarcinoma. *Oncotarget* 6 25897–25916. 10.18632/oncotarget.4532 26317790 PMC4694874

[B28] Nakajima M. Kato H. (2013). Treatment options for esophageal squamous cell carcinoma. *Expert Opin. Pharmacother* 14 1345–1354. 23675862 10.1517/14656566.2013.801454

[B29] Odaka Y. Xu B. Luo Y. Shen T. Shang C. Wu Y. (2014). Dihydroartemisinin inhibits the mammalian target of rapamycin-mediated signaling pathways in tumor cells. *Carcinogenesis* 35 192–200. 10.1093/carcin/bgt277 23929438 PMC3871936

[B30] Qin G. Zhao C. Zhang L. Liu H. Quan Y. Chai L. (2015). Dihydroartemisinin induces apoptosis preferentially via a Bim-mediated intrinsic pathway in hepatocarcinoma cells. *Apoptosis* 20 1072–1086. 10.1007/s10495-015-1132-2 25935454

[B31] Rimkus T. K. Carpenter R. L. Qasem S. Chan M. Lo H. W. (2016). Targeting the sonic hedgehog signaling pathway: review of smoothened and GLI inhibitors. *Cancers (Basel)* 8:22. 10.3390/cancers8020022 26891329 PMC4773745

[B32] Rubenstein J. H. Shaheen N. J. (2015). Epidemiology, diagnosis, and management of esophageal adenocarcinoma. *Gastroenterology* 149 302–317. 10.1053/j.gastro.2015.04.053 25957861 PMC4516638

[B33] Ruiz i Altaba A. Sánchez P. Dahmane N. (2002). Gli and hedgehog in cancer: tumours, embryos and stem cells. *Nat. Rev. Cancer* 2 361–372. 10.1038/nrc796 12044012

[B34] Sims-Mourtada J. Izzo J. G. Ajani J. Chao K. S. (2007). Sonic Hedgehog promotes multiple drug resistance by regulation of drug transport. *Oncogene* 26 5674–5679. 10.1038/sj.onc.1210356 17353904

[B35] Sugimura K. Miyata H. Tanaka K. Hamano R. Takahashi T. Kurokawa Y. (2012). Let-7 expression is a significant determinant of response to chemotherapy through the regulation of IL-6/STAT3 pathway in esophageal squamous cell carcinoma. *Clin. Cancer Res.* 18 5144–5153. 10.1158/1078-0432.ccr-12-0701 22847808

[B36] Wang L. Chen G. Chen K. Ren Y. Li H. Jiang X. (2015). Dual targeting of retinoid X receptor and histone deacetylase with DW22 as a novel antitumor approach. *Oncotarget* 6 9740–9755. 10.18632/oncotarget.3149 25762635 PMC4496394

[B37] Wang L. H. Li Y. Yang S. N. Wang F. Y. Hou Y. Cui W. (2014). Gambogic acid synergistically potentiates cisplatin-induced apoptosis in non-small-cell lung cancer through suppressing NF-κB and MAPK/HO-1 signaling. *Br. J. Cancer* 110 341–352. 10.1038/bjc.2013.752 24300974 PMC3899775

[B38] Wang Y. F. Chang C. J. Lin C. P. Chang S. Y. Chu P. Y. Tai S. K. (2012). Expression of hedgehog signaling molecules as a prognostic indicator of oral squamous cell carcinoma. *Head Neck.* 34 1556–1561. 10.1002/hed.21958 22287313

[B39] Xie J. Murone M. Luoh S. M. Ryan A. Gu Q. Zhang C. (1998). Activating smoothened mutations in sporadic basal-cell carcinoma. *Natur* 391 90–92. 10.1038/34201 9422511

[B40] Yamasaki M. Makino T. Masuzawa T. Kurokawa Y. Miyata H. Takiguchi S. (2011). Role of multidrug resistance protein 2 (MRP2) in chemoresistance and clinical outcome in oesophageal squamous cell carcinoma. *Br. J. Cancer* 104 707–713. 10.1038/sj.bjc.6606071 21206495 PMC3049584

[B41] Yang L. Ren Y. Yu X. Qian F. Bian B. S. Xiao H. L. (2014). ALDH1A1 defines invasive cancer stem-like cells and predicts poor prognosis in patients with esophageal squamous cell carcinoma. *Mod. Pathol.* 27 775–783. 10.1038/modpathol.2013.189 24201124

[B42] Yang L. Wang L. S. Chen X. L. Gatalica Z. Qiu S. Liu Z. (2012). Hedgehog signaling activation in the development of squamous cell carcinoma and adenocarcinoma of esophagus. *Int. J. Biochem. Mol. Biol.* 3 46–57. 22509480 PMC3325770

[B43] Yoo Y. A. Kang M. H. Lee H. J. Kim B. H. Park J. K. Kim H. K. (2011). Sonic hedgehog pathway promotes metastasis and lymphangiogenesis via activation of Akt, EMT, and MMP-9 pathway in gastric cancer. *Cancer Res.* 71 7061–7070. 10.1158/0008-5472.can-11-1338 21975935

[B44] Zahreddine H. A. Culjkovic-Kraljacic B. Assouline S. Gendron P. Romeo A. A. Morris S. J. (2014). The sonic hedgehog factor GLI1 imparts drug resistance through inducible glucuronidation. *Nature* 511 90–93. 10.1038/nature13283 24870236 PMC4138053

