# Supplementary figures and images for: Dihydroartemisinin Sensitizes Esophageal Squamous Cell Carcinoma to Cisplatin by Inhibiting Sonic Hedgehog Signaling
Source: Front Cell Dev Biol. 2020 Dec 10;8:596788. doi: 10.3389/fcell.2020.596788 (PMC7758349; doi:10.3389/fcell.2020.596788)

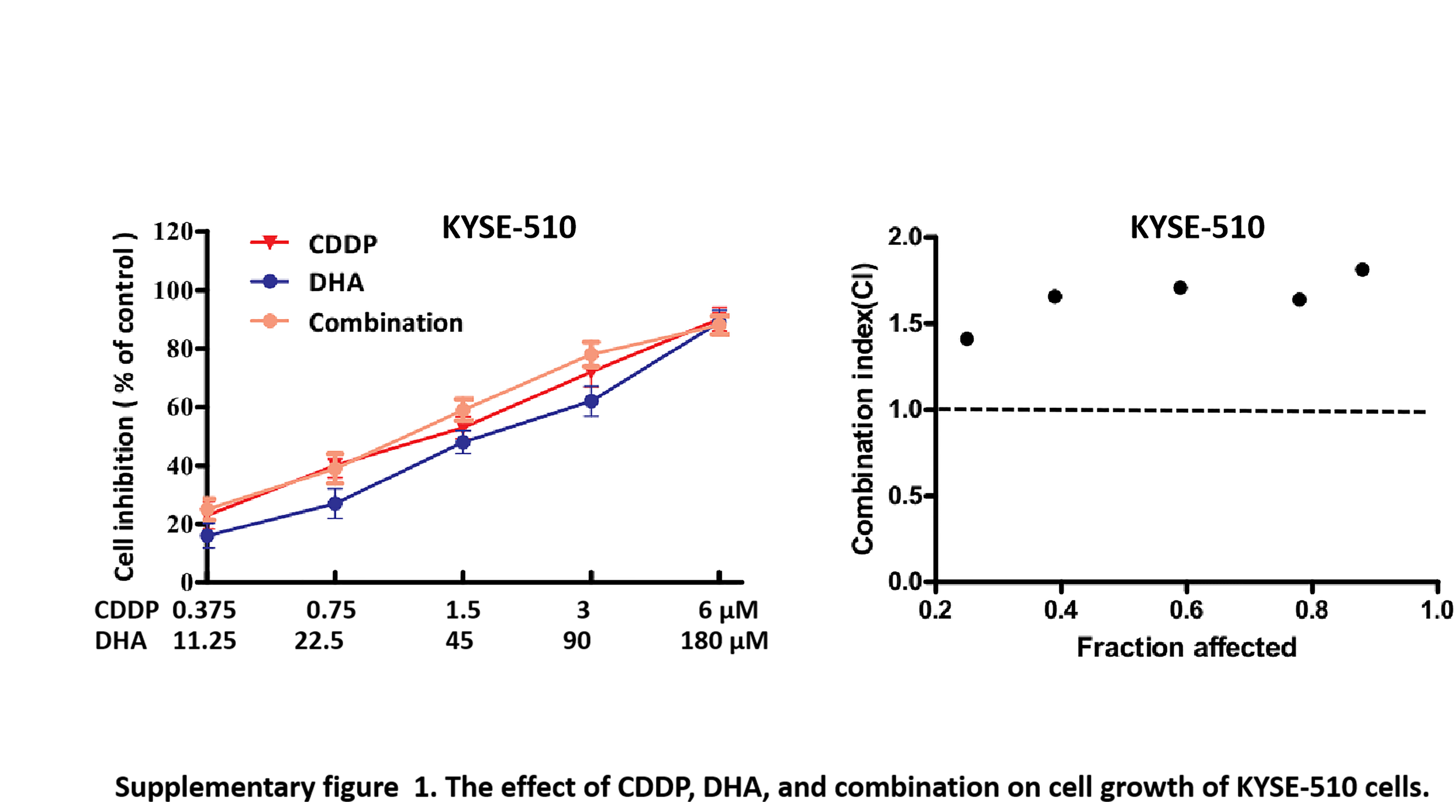

Supplement: Supplementary Figure 1 — The effect of CDDP, DHA, and combination on cell growth of KYSE-510 cells. [file Image_1.JPEG]

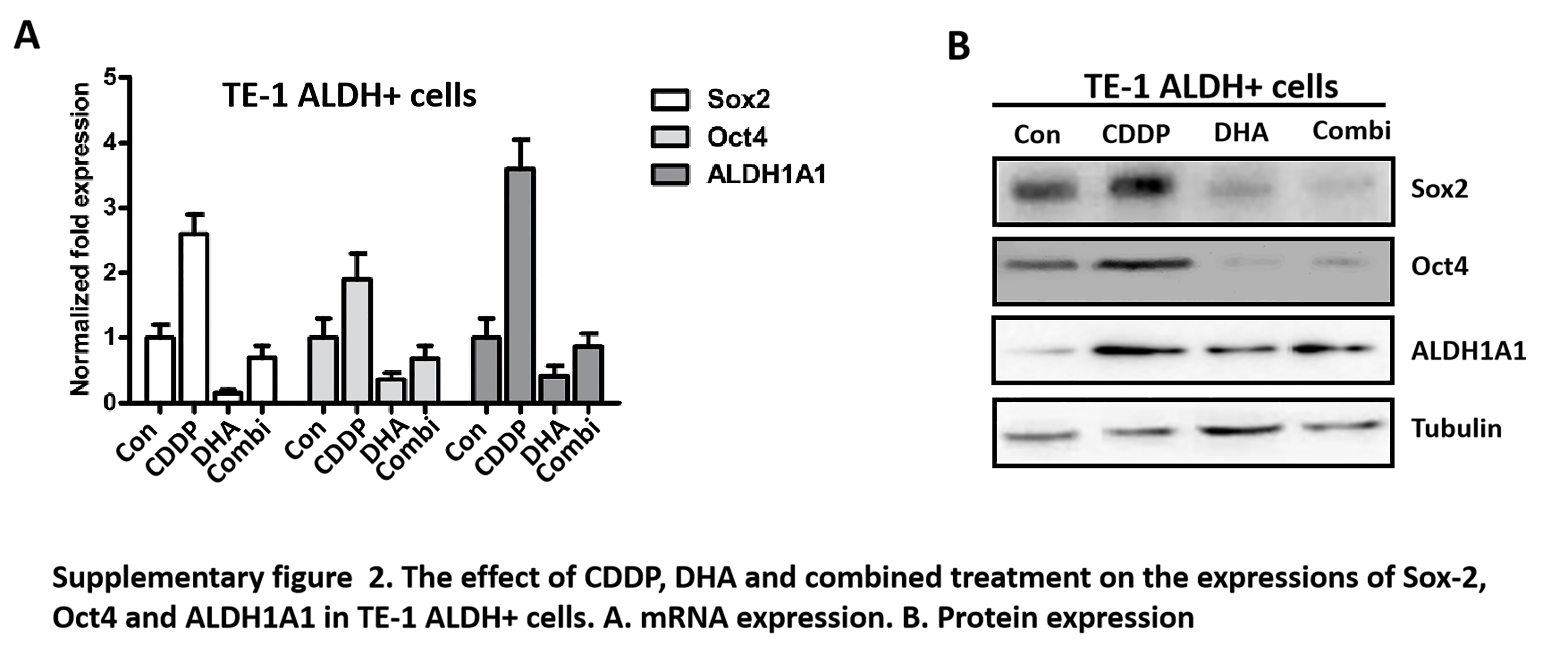

Supplement: Supplementary Figure 2 — The effect of CDDP, DHA, and combined treatment on the expressions of Sox-2, Oct4, and ALDH1A1 in TE-1 ALDH + cells. [file Image_2.JPEG]
