# Supplementary material for: Dihydroartemisinin Sensitizes Esophageal Squamous Cell Carcinoma to Cisplatin by Inhibiting Sonic Hedgehog Signaling
Source: Front Cell Dev Biol. 2020 Dec 10;8:596788. doi: 10.3389/fcell.2020.596788 (PMC7758349; doi:10.3389/fcell.2020.596788)
Supplement: Supplementary Table 1 — The clinicopathological parameters of ESCC patients. [file Table_1.DOCX]

**Table S1. The clinicopathological parameters of ESCC patients．**

| **Variable** | **N** |
| --- | --- |
| **Age** |  |
| < 60 years | 32 |
| ≥ 60 years | 27 |
| **Gender** |  |
| Male | 48 |
| Female | 11 |
| **T stage** |  |
| I | 15 |
| II | 27 |
| III | 14 |
| IV | 2 |
| **Lymph node metastasis** |  |
| Presence | 15 |
| Absence | 44 |
| **Chemotherapy** |  |
| Cisplatin | 9 |
| Oxaliplatin | 41 |
| Lobaplatin | 8 |
| Carboplatin | 1 |
