# Supplementary material for: Dihydroartemisinin Sensitizes Esophageal Squamous Cell Carcinoma to Cisplatin by Inhibiting Sonic Hedgehog Signaling
Source: Front Cell Dev Biol. 2020 Dec 10;8:596788. doi: 10.3389/fcell.2020.596788 (PMC7758349; doi:10.3389/fcell.2020.596788)
Supplement: Supplementary Table 2 — The information for antibodies used in this study. [file Table_2.DOCX]

**Table S2.**

**The information for antibodies used in this study.**

| **Protein** | **Catlog#** | **Colony** | **Isotype** | **Company** |
| --- | --- | --- | --- | --- |
| Shh | ab53281 | Monoclonal | Rabbit | Abcam |
| PTCH1 | ab53715 | Polylonal | Rabbit | Abcam |
| Gli1 | ab49314 | Polylonal | Rabbit | Abcam |
| Sox2 | ab93689 | Monoclonal | Rabbit | Abcam |
| Nanog | 4903S | Monoclonal | Rabbit | CST |
| Oct4 | 2890S | Monoclonal | Rabbit | CST |
| P-gp | 12683S | Monoclonal | Rabbit | CST |
| ALDH1A1 | 36671S | Monoclonal | Rabbit | CST |
| Tubulin | 2144S | Polylonal | Rabbit | CST |
| β-actin | 4970S | Monoclonal | Rabbit | CST |
