# Supplementary material for: Dihydroartemisinin Sensitizes Esophageal Squamous Cell Carcinoma to Cisplatin by Inhibiting Sonic Hedgehog Signaling
Source: Front Cell Dev Biol. 2020 Dec 10;8:596788. doi: 10.3389/fcell.2020.596788 (PMC7758349; doi:10.3389/fcell.2020.596788)
Supplement: Supplementary Table 3 — The primer sequences for qRT-PCR analyses. [file Table_3.DOCX]

**Table S3**

**The primer sequences for qRT-PCR analyses**

| **Genes** | **Primer sequences** | |
| --- | --- | --- |
| **Gli1** | **F: AGCGTGAGCCTGAATCTGTG** | **R: CAGCGATGTACTGGGCTTTGAA** |
| **MDR1** | **F: TTGCTGCTTACATTCAGGTTTCA** | **R: AGCCTATCTCCTGTCGCATTA** |
| **PTCH1** | **F: AAGAAGGTGCTAATGTCCTGAC** | **R: GTCCCAGACTGTAATTTCGCC** |
| **OCT4** | **F: GCAGCGACTATGCACAACGA** | **R: CCAGAGTGGTGACGGAGACA** |
| **SOX2** | **F: CATCACCCACAGCAAATGACA** | **R: GCTCCTACCGTACCACTAGAACTT** |
| **GAPDH** | **F：TTCCTCTTGTGCTCTTGCTGG** | **R：CCCTCAACGACCACTTTGTCA** |
